# Supplementary material for: Evaluation of Vertebrate-Specific Replication-Defective Zika Virus, a Novel Single-Cycle Arbovirus Vaccine, in a Mouse Model
Source: Vaccines (Basel). 2021 Apr 1;9(4):338. doi: 10.3390/vaccines9040338 (PMC8065927; doi:10.3390/vaccines9040338)
Supplement: Supplementary file 1 [file vaccines-09-00338-s001.pdf]

The cytokine responses in the pregnant mouse brain

Five-week-old female AG129 mice were immunized with 1×10^6 FFU VSRD-ZIKV, or DMEM through the s.c. route. On day 14 post-immunization (p.i). the 1×10^6 FFU VSRD-ZIKV group was immunized with 1×10^6 FFU VSRD-ZIKV again. The female AG129 mice received a prime-boost regimen of VSRD-ZIKV were mated on day 22 post-infection. The pregnant mice were then challenged with a lethal ZIKV infection (PRAVABC59 strain) and the placenta, fetal brain, and pregnant mouse brain were assessed 12 days post-infection for cytokine expression and histopathology (Figure, 4). Total RNA of cells was extracted by Trizol reagent (Invitrogen), and 1 μg of RNA was used to synthesize cDNA using a first strand cDNA synthesis kit of SuperScript III System (ThermoFisher Scientific, Waltham, MA). Quantitative real-time RT-PCR was performed using a 7500 real-time PCR system (Applied Biosystems) and SYBR green PCR master mix (Toyobo, Osaka, Japan). Data were normalized to the level of β-actin expression in each sample. Primers used are listed in Table S1. The relative expression of TNF-α, CCL-5, IL-1β, and IL-6 were normalized to the levels of endogenous control β-actin within each sample using the $2{-}\Delta\Delta CT$ (where CT is threshold cycle) method.

Table S1. Primer pairs used for quantitative real-time RTPCR analyses.

Primer name	Sequence (5'-3')
β-actin-F	CACTGCCGCATCCTCTTCCTCCC
β-actin-R	CAATAGTGATGACCTGGCCGT
TNF-a-F	TGTCTCAGCCTCTTCTCATTCC
TNF-a-R	TTAGCCCACTTCTTTCCCTCAC
CCL-5-F	TGCCCACGTCAAGGAGTATTTC
CCL-5-R	AACCCACTTCTTCTCTGGGTTG
IL-6-F	CTGCTTCTGGTGATGGCTACTG
IL-6-R	GGCATCACCTTTGGCATCTT
IL-1β-F	AACCTGCTGGTGTGTGACGTTC
IL-1β-R	CAGCACGAGGCTTTTTTGTTGT

Notes: F for forward primers, R for reverse primers.

Figure S1. The cytokine responses in the pregnant mouse brain.

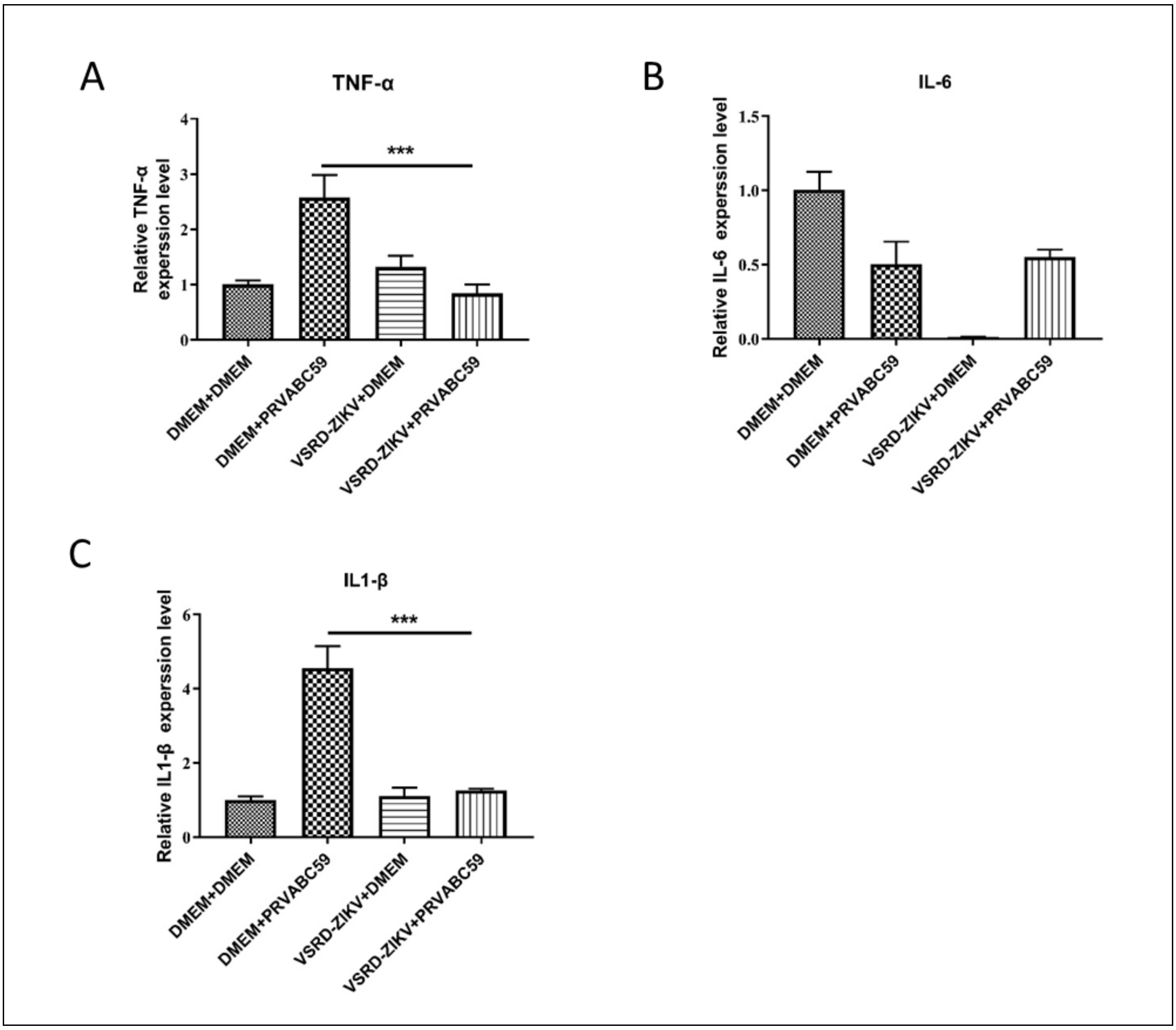

Figure, S1. The cytokine responses in the pregnant mouse brain. Five-week-old female AG129 mice (n = 9) were immunized with 1 × 10^6 FFU VSRD-ZIKV, or DMEM through the s.c. route. On day 14 p.i. the 1 × 10^6 FFU VSRD-ZIKV group were immunized with 1 × 106 FFU VSRD-ZIKV again. On day 22 p.i. immunized AG129 female mice were mated with naïve male AG129 mice. At E6, mice were inoculated subcutaneously with 10^3 FFU of ZIKV PRVABC59 via intraperitoneal injection. All animals were sacrificed on E18 and the cytokine responses in the pregnant mouse brain were assessed by qRT-PCR: (A) TNF-α; (B) IL-6; and (c) IL1-β. Data are expressed as means ± SEM from three independent experiments. ***$P < 0.001$.
